# Soft Electronics Enabled Ergonomic Human-Computer Interaction for Swallowing Training

**DOI:** 10.1038/srep46697

**Published:** 2017-04-21

**Authors:** Yongkuk Lee, Benjamin Nicholls, Dong Sup Lee, Yanfei Chen, Youngjae Chun, Chee Siang Ang, Woon-Hong Yeo

**Affiliations:** 1 Department of Mechanical and Nuclear Engineering, School of Engineering, Virginia Commonwealth University, Richmond, VA 23284, USA; 2 School of Engineering and Digital Arts, University of Kent, Canterbury, United Kingdom; 3 Department of Industrial Engineering, University of Pittsburgh, Pittsburgh, PA 15261, USA; 4 Department of Bioengineering, University of Pittsburgh, Pittsburgh, PA 15261, USA; 5 Center for Rehabilitation Science and Engineering, School of Medicine, Virginia Commonwealth University, Richmond, VA 23298, USA

## Abstract

We introduce a skin-friendly electronic system that enables human-computer interaction (HCI) for swallowing training in dysphagia rehabilitation. For an ergonomic HCI, we utilize a soft, highly compliant (“skin-like”) electrode, which addresses critical issues of an existing rigid and planar electrode combined with a problematic conductive electrolyte and adhesive pad. The skin-like electrode offers a highly conformal, user-comfortable interaction with the skin for long-term wearable, high-fidelity recording of swallowing electromyograms on the chin. Mechanics modeling and experimental quantification captures the ultra-elastic mechanical characteristics of an open mesh microstructured sensor, conjugated with an elastomeric membrane. Systematic *in vivo* studies investigate the functionality of the soft electronics for HCI-enabled swallowing training, which includes the application of a biofeedback system to detect swallowing behavior. The collection of results demonstrates clinical feasibility of the ergonomic electronics in HCI-driven rehabilitation for patients with swallowing disorders.

Dysphagia, a swallowing disorder, refers to difficulty or impossibility in swallowing food or liquid. This disease emerges from a variety of medical conditions including stroke/brain injury^1^,^2^,^3^, Parkinson’s disease^4^,^5^, cancer^6^,^7^,^8^, and gastroesophageal reflux disease^9^. Dysphagia can occur at any age, but it is more common in the elderly^10^,^11^,^12^. The potential health risks associated with dysphagia include dehydration, malnutrition, aspiration pneumonia, and death^13^,^14^. In addition, dysphagia can negatively affect all aspects of life; patients may need assistance with eating or feel embarrassed during mealtime, which results in avoidance of social activities or reductions in patients’ dignity and self-esteem^15^. Various methods, such as medications, dietary modification, surgery, and exercise rehabilitation, have been used to treat patients with dysphagia^16^,^17^,^18^. Particularly, exercise-based rehabilitation has been served as an ideal method by restoring their independence and quality of life. A few examples of dysphagia rehabilitation are changing swallowing motions^19^, strengthening muscles through exercise^20^,^21^, and stimulating the sensory system^22^. Recent studies on human-computer interaction (HCI) systems^23^,^24^, offering direct communication pathways between human and external devices, have demonstrated more efficient treatments by helping patients to monitor and adjust swallowing patterns via exercises^25^,^26^,^27^. Most of non-invasive HCI systems utilize a wide range of human physiological signals such as electromyograms (EMG), electrocardiograms (ECG), and electroencephalograms (EEG) to offer direct user interfaces^28^,^29^ and assist people with certain disabilities^30^,^31^,^32^,^33^. Thus, continuous, uninterrupted acquisition of high-fidelity physiological signals serves as a key requirement for a successful HCI.

Conventional, non-invasive measurement of electrophysiology on the skin incorporates surface-mounted electrodes with adhesive pads and conductive gels, which constraints natural skin deformation and even causes adverse effects like skin irritation and allergic reactions^34^. To address those issues, our prior works^35^,^36^,^37^ introduced conformal electronic systems that are ultra-thin, ultra-light, and deformable, while minimizing thermal and mechanical loadings to the skin. The small form factor of the soft electrodes has offered intimate integration to the skin and demonstrated various applications for long-term recording of EMG^38^, ECG^39^, and EEG^36^, temperature mapping^37^, thermal conductivity^35^, hydration^40^, and skin-like stimulation^41^. A similar setting was used in a recent study^42^ for swallowing EMG detection by comparing the functionality with conventional medical electrodes. This work, however, only included a pilot study of signal measurement capabilities. Moreover, the electrode stretchability is limited in 30%, which would cause failure issues in the clinical use with patients since multiple reports claimed the skin extensibility is higher than 30% and even up to 70%^43^,^44^,^45^,^46^.

In this paper, we expand the idea of using ultra-stretchable (>100%), skin-wearable, conformal electrodes to design an ergonomic HCI system for swallowing training in dysphagia rehabilitation. Figure 1 shows the overview of a HCI training interface for swallowing exercise by using “skin-like” electrodes, laminated onto the curvilinear surface of the submental muscles on the chin. The HCI training system offers a game-based biofeedback based on swallowing EMG signals. A classification algorithm detects the targeted swallowing activities in a binary manner using a threshold-based technique, which is utilized to simultaneously control an object (“jumping” ball between moving plates) in the training game. Computational mechanics study establishes fundamental design guidelines for an ultra-flexible and stretchable electrode, resulting in conformal contact for high-fidelity recording of surface EMG without the use of conductive gels. Direct comparison between skin-like and conventional rigid electrodes in swallowing measurement captures the advantage of the soft electronics for ergonomic HCI systems. Collectively, this work demonstrates the feasibility of the skin-like electronics-enabled, user-comfortable HCI as a rehabilitation tool.

## Results

### Design and fabrication of a skin-like electrode

**Figure 2:**
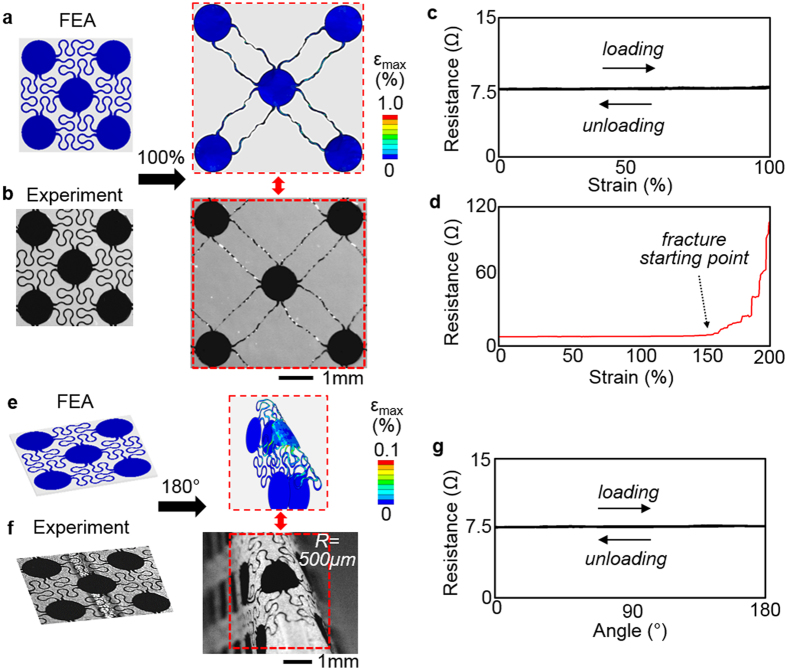
Computational modeling and experimental quantification of a fractal structured electrode upon mechanical deformation. (**a**,**b**,**e**,**f**) Comparison between the FEA results and experimental observations with (**a**,**b**) biaxial tensile stretching upto 100% and (**e**,**f**) bending upto 180 degrees with the radius of curvature of R = 500 μm. (**c**,**d**,**g**) Quantification of electrical resistance according to the (**c**,**d**) applied strains and (**g**) bending; (**c**) cyclic mechanical test with repetition of loading and unloading, (**d**) continuous loading with 10% strain increment to reveal fracture points, and (**g**) cyclic mechanical bending test. The scale bars in the FEA data indicate the maximum principal strain applied on the electrode.

We designed a soft, ultra-stretchable electrode to realize a comfortable, intimate contact on the sensitive skin area like the neck. A finite element analysis (FEA), shown in Fig. 2a and e, determines key parameters to design a mechanically compliant structure. For applications of the device with human subjects, the electrode needs to accommodate mechanical strain over 70% by considering the maximum skin extensibility, device cleaning, and handling factors^43^,^44^,^45^,^46^,^47^. The entire structure of an electrode is composed of self-similar patterns such as fractal curves^39^, which yield superior elastic mechanical properties, compared to conventional filamentary serpentine patterns. The computational study estimates the maximum mechanical principal strains on the electrode design, caused by skin deformation during time-dynamic movement (bending and stretching) on the skin.

The computational results in Fig. 2a and e show estimated mechanical behavior of the fractal interconnected circular cells under applied strains and bending. The entire area of a skin-wearable electrode is 1 cm^2^ and the width of each fractal curve and radius of a circular cell are 50 μm and 500 μm, respectively (details in Supplementary Figure S1). A biocompatible Au nanomembrane (300 nm in thickness), supported by thin layers of polyimide and elastomeric membrane, makes the direct contact to the skin for surface EMG recording. The FEA modeling calculates the maximum principal strains^48^ on the nanomembrane (fracture strain of Au: 5%). The fractal electrode shows a highly elastic mechanical behavior, even with more than 100% of biaxial tensile strains (Fig. 2a; calculated maximum principal strain <1%), which is validated by both experimental observation (Fig. 2b) and electrical resistance measurement (Fig. 2c and d). An automated mechanical stretcher applies a cyclic loading-unloading onto a sample electrode with 10% of strain increment, which monitors the elastic behavior (details of the experimental setup in Supplementary Figure S2). The complete fracture is observed when the applied strain reaches at ~200% (Fig. 2d), while a localized fracture starts to occur at ~150%, detected by both optical imaging and electrical measurement (Supplementary Figure S2). The fractal-structured electrode shows negligible effects upon cyclic bending up to 180 degrees with the radius of curvature of 500 μm, estimated by the FEA result (Fig. 2e) and validated by the experiment (Fig. 2f). The corresponding electrical recording of the bending effect (Fig. 2g) measures the resistance change smaller than 0.15 Ω (Supplementary Figure S3). Overall, the quantitative computational and experimental studies of the structural mechanics prove that the fractal-inspired, ultra-deformable structure can be safely applicable on the skin.

**Figure 3:**
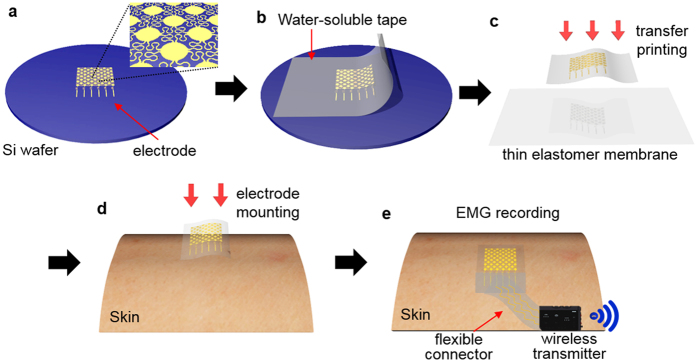
Fabrciation process of a skin-like electrode and printing on the skin. (**a**) Fabricated gold electrode on a Si wafer. (**b**) Retrieved electrode from the wafer by usig a water-soluble tape. (**c**) Tranfer printing onto a thin elastomeric membrane by dissolving the tape with water. (**d**) Printing of the electrode on the target location of the skin by dissolving a supporting sheet of the polyvinylalcohol film. (**e**) Connection of a ribbon cable for wireless transmission of the recorded EMG signals.

Schematic illustrations in Fig. 3 describe the fabrication process of an electrode based on the combination of a wafer-scale microfabrication and material transfer printing (details in Method section and Supplementary Note 1). A 3-inch silicon wafer serves as a temporary carrier to build an electronic component: Au nanomembrane having a series of fractal interconnects and circular cells (Fig. 3a). Dissolving a sacrificial polymer, poly(methyl methacrylate), and attachment of a water soluble tape retrieves the fabricated electrode from the carrier (Fig. 3b). The first transfer printing onto a 5 μm-thick elastomeric membrane is facilitated by a covalent bonding (Si – O – Si) with a deposition of a SiO_2_ layer on the electrode (Fig. 3c). Laminating the electrode by dissolving a water soluble carrier onto the skin (Fig. 3d and e) is the last step to measure surface EMG signals. Note that a flexible, microscale ribbon cable connects the electrode to a wireless data transmitter (Supplementary Figure S4). The recorded analogue EMG signals from the skin are digitally converted, amplified, and wirelessly transmitted to an external receiver via a Bluetooth system (BioRadio; Great Lakes NeuroTechnologies, Cleveland, OH). An electrode supported by an ultrathin elastomeric membrane finds the best use for long-term wearing more than a week, facilitated by the use of spray-on-bandage and releasable connector^37^. The main advantage of the skin-like electrode lies on the capability of gentle, reversible contact to the skin without the use of gels, facilitated by the lowered bending stiffness and open mesh meander traces.

### Performance comparison between conventional rigid- and skin-like electrodes

**Figure 4:**
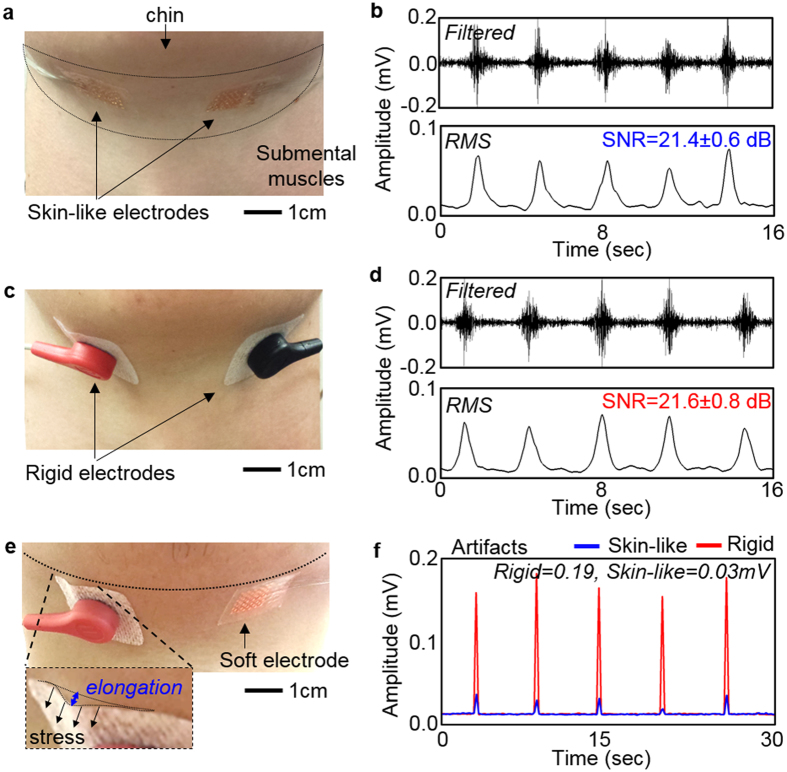
Performance comparison between gel-based rigid and skin-like electrodes. (**a**,**c**) Photos of paired electrodes mounted on the submental muscles; (**a**) skin-like electrodes mounted by an elastomeric membrane and (**c**) rigid electrodes mounted by an adhesive pad. (**b**,**d**) Swallowing EMG signals (top graph: filtered data and bottom graph: RMS data), recorded by (**b**) skin-like electrodes and (**d**) rigid electrodes. (**e**) Significantly elongated tissue by the applied stress from the rigid electrode, while the soft electrode shows a negligible effect. (**f**) Comparison plot showing the issue of the rigid electrode with substantial motion artifacts (~6 times higher than the soft electrode).

In this study, we make a quantitative assessment of clinical feasibility of the skin-like electronics via the performance comparison with a conventional medical-grade electrode (MVAP II snap electrode; MVAP Medical Supplies, Newbury Park, CA). Qualitative assessment of the device comfort appears in the “*in vivo* evaluation” section. For the performance comparison, EMG signals from swallowing activities were recorded separately from each set of rigid electrodes and skin-like electrodes on a subject. Both electrodes were placed on the same location of the submental muscles. Figure 4 summarizes the recorded EMG signals for performance comparison. The swallowing EMG recording requires a pair of electrodes, mounted in the horizontal direction by aligning with the submental muscles, for differential recording: one for recording and the other for reference (Fig. 4a and c). The ground electrode is located on the forehead to remove signals from the common, inherent body induced noise. The tested exercise used liquid swallowing that is one of common compensatory strategies for oropharyngeal dysphagia^49^. A subject periodically drinks a sip of water (20 mL) at every 3 seconds to include 10 trials. All EMG data in Fig. 4 have a sampling rate of 1 kHz. Four plots in Fig. 4b and d show a set of representative results of swallowing EMG signals from skin-like and rigid electrodes, respectively. The measured data were bandpass filtered from 30 to 150 Hz and further smoothed for root-mean-square (RMS) signals using a windowed average (250 samples). The highest signal peaks were observed during laryngeal elevation with limited the cross talk from other muscles^50^,^51^. Calculation of signal-to-noise (SNR) numbers provides quantitative comparison between two different electrodes. SNR values are calculated by:


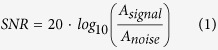


where *A* is signal amplitude from RMS values. ‘Signal’ values are from the swallowing activity while ‘noise’ comes from no activity period (details in Supplementary Figure S5). The averaged SNR values from 10 different trials show negligible difference with 21.4 ± 0.6 dB and 21.6 ± 0.8 dB for skin-like and rigid electrodes, respectively. The result demonstrates the capability of the skin-like electrodes, offering highly conformal lamination onto the curvilinear skin, even without electrolyte gels.

In addition, the experimental study captures a key advantage of the soft electrode. The rigid electrode, in conjunction with an adhesive pad and gel, applies excessive stresses to the soft skin on the chin area (Fig. 4e). The effective weight of the rigid electrode is 4.5 g, 75 times higher than that of the soft electrode (0.06 g). The elongated amount (strain) of the skin in Fig. 4e due to the applied stress can be calculated by:


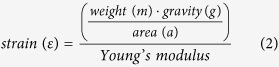


where Young’s modulus of the skin is 25–100 kPa^52^, which varies depending on body site, *g* = 9.8 m/s^2^, and *a* is 1 cm^2^ for both electrodes. Assuming the Young’s modulus of loose neck skin is 25 kPa, the calculated strains are 1.77% and 0.02% for the rigid and the skin-like electrode, respectively. While the negligible strain from the skin-like electrode assures its conformal interface to the skin, the rigid electrode excessively deforms the skin surface, which is closely related to the sensitivity to external motion artifacts. Motion artifacts are shown as low frequency components, originated from the electrode-skin interface^53^. Therefore, intimate and conformal contact of the electrode to the skin is critical to minimize the motion artifacts. Applied periodic pressure to a localized site on the chin creates parasitic effects in the EMG signal measurement. The associated motion artifacts are measured and compared in Fig. 4f, which clearly show that the soft electrode interface has a negligible effect to the skin deformation (peak average: 0.03 ± 0.01 mV), compared with the conventional one (0.19 ± 0.01 mV). The significant difference may come from the conformability of the soft electrode to accommodate the skin motions without relative motion at the skin-electrode interface, while the rigid electrode including the gel has such unfavorable motion. The motion artifacts had a negative effect on the classification by showing a higher false positive rate for the rigid electrode (5%) than the skin-like electrode (3%). Details are shown in Table 1.

### Development of a data acquisition and biofeedback interface

**Figure 5:**
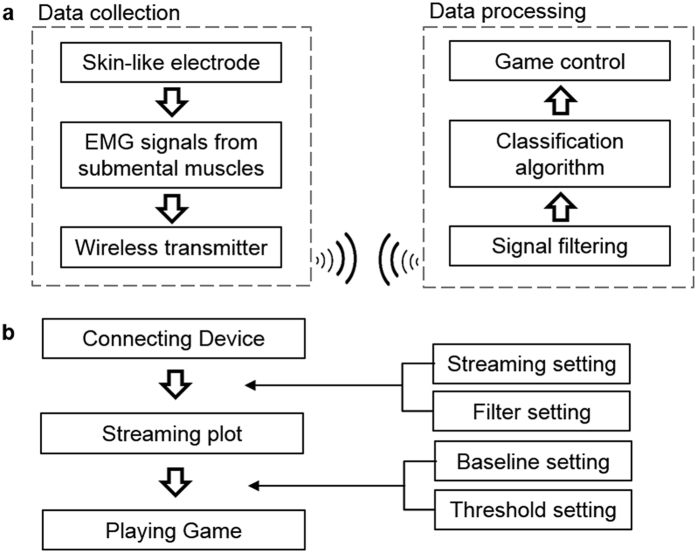
Data acquisition system and biofeedback interface. (**a**) Flow chart describing the entire process from data acquisition to signal processing. (**b**) Flow chart of the real-time biofeedback interface for a HCI control. A series of parameters including filters and thresholds are adjustable for individual calibration.

A game-based HCI, driven by swallowing EMG activities, was developed to demonstrate the use of an ergonomic HCI for training. The HCI development includes three components: 1) development of data acquisition interface for detection of swallowing EMG signals on the chin, 2) development of signal classification algorithm, and 3) development of a real-time game control to get a biofeedback. A schematic diagram in Fig. 5a shows the sequential steps from data collection to processing and game control. A set of skin-like electrodes, mounted on a subject’s submental muscles, records targeted EMG signals from swallowing activities via a flexible ribbon cable, connected to a Bluetooth-based wireless transmitter (details in Supplementary Figure S4a–S4c). A wireless USB dongle, connected to a computer interface, receives the data to distinguish via classification algorithm and control the biofeedback game. A control interface, programmed by C# and designed by Microsoft. NET Framework 4.5, monitors the status of the device, data recording, and parameter settings (streaming, filter, and threshold) for signal classification (Fig. 5b and Supplementary Information S4d).

**Figure 6:**
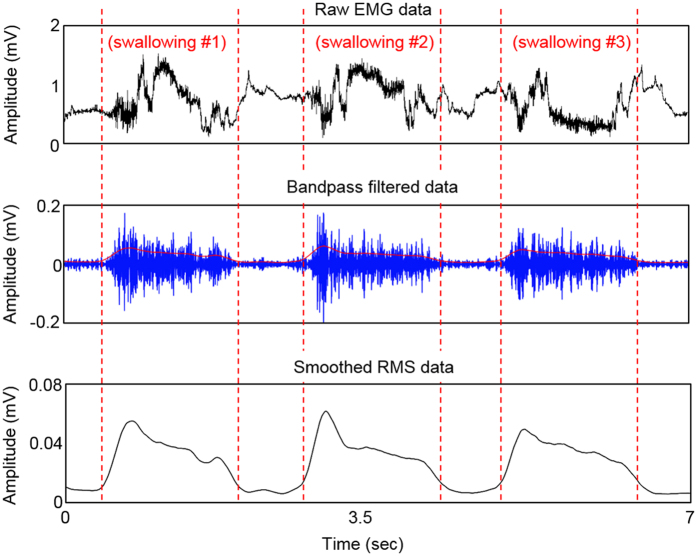
Processing flow of swallowing EMG signals. Three plots show a set of representative swallowing detection; raw EMG data (top), bandpass filtered data (middle), and smoothed RMS data (bottom).

Figure 6 captures a representative set of EMG signals recorded from swallowing activities. The recorded data from a subject are processed via a filtering process, designed by Matlab (MathWorks, Natick, MA). For example, a periodic activity of dry swallowing provides a series of amplitude change (mV) according to time (top graph in Fig. 6). The raw data are then bandpass filtered at the frequencies of 30 and 150 Hz to eliminate high frequency noise and motion artifacts^54^ (middle graph in Fig. 6). Afterwards, the signals are further smoothed by taking root-mean-square (RMS) values based on the signal window of 250 samples (bottom graph in Fig. 6), which provides a clear informative interpretation. The RMS values then go through the classification algorithm to distinguish active segments from swallowing activities.

**Figure 7:**
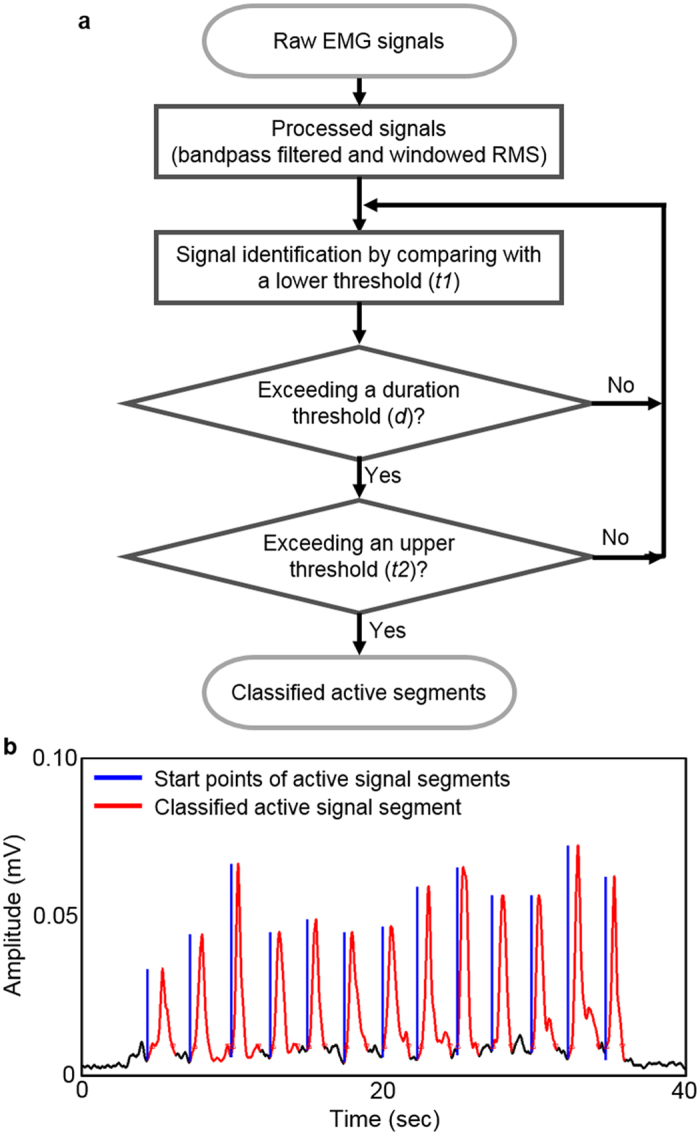
Data classification algorithm. (**a**) Flow chart describing a classification algorithm with a three-part threshold technique. (**b**) Plot of a classification result for swallowing EMG signals obtained from skin-like electrodes.

The flow chart in Fig. 7a summarizes the sequential steps from the acquisition of raw signals (swallowing EMG) to data classifications by following the developed algorithm (detail description in Supplementary Figure S6). In this work, we used a three-part threshold technique to identify the swallowing activities, which was included in the designed game-control system with biofeedback algorithm. By considering the nature of EMG signal patterns from swallowing activities, we found that three-part threshold method offered better classification performance than a single-threshold technique (detail information in Supplementary Table S1). The main advantage of the threshold-based method is the ability to offer a quick data calibration and short computation time for real-time HCI, but a limitation is to distinguish swallowing from other muscular activities.

The presented threshold method consists of two magnitude-based thresholds and a duration-based threshold. When a period of swallowing EMG signals exceed those three thresholds, it is classified as an active signal segment. While the duration threshold (*d*) was set to 0.1 seconds (100 samples) and kept constant for all subjects during user evaluation, the two threshold values, lower (*t1*) and upper (*t2*) magnitude threshold values, needed to be calibrated individually for each subject after reviewing the signals due to physiological differences between subjects^51^. At the first, the magnitude analysis detects a period of the recorded EMG signals where their magnitudes exceed the lower magnitude threshold. When the period of the detected EMG signals satisfies the duration threshold, the magnitude analysis examines the period of the detected EMG signals again to check if the EMG signals include a peak magnitude exceeding the upper magnitude threshold. Once the period of swallowing EMG signals is classified as the active signal segment, the end point of the active signal segment is determined where the active signal segment drops back below the lower magnitude threshold. It is found that the three-part threshold technique achieves reasonable classification rates when the two magnitude thresholds are carefully adjusted. A graph in Fig. 7b summarizes the data classification result using the three-part threshold technique for liquid swallowing EMG, collected from skin-like electrodes. Performance comparison with rigid electrodes is shown in Supplementary Figure S7. During the classification, the two magnitude thresholds, lower and upper magnitudes, provide 100% classification rates for given recorded swallowing EMG signals. The result suggests that most of swallowing activities can be easily distinguishable with the developed classification algorithm.

**Figure 8:**
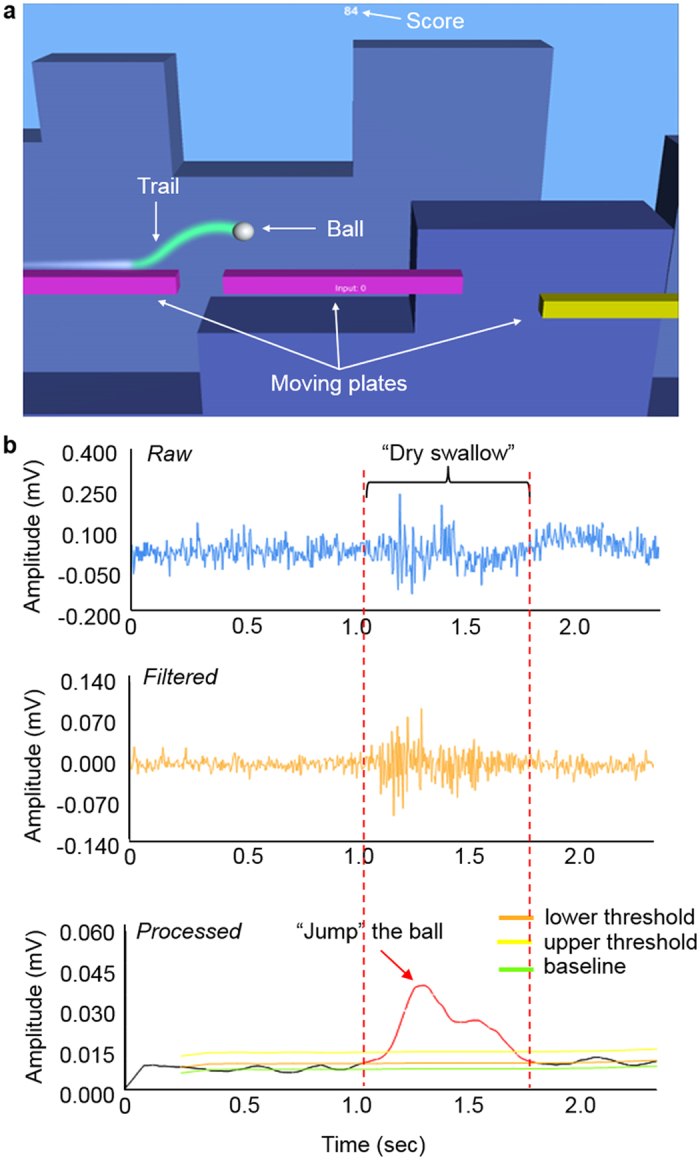
HCI for swallowing training: biofeedback game. (**a**) Screenshot of the biofeedback game, controlled by swallowing EMG signals. Binary data from the main application indicate activity or no activity, which are used in the game to jump the white ball. (**b**) Real-time display plots showing raw, filtered, and processed signals. The processed signals represent active signal segments according to the thresholds.

The active signal segments, determined by the classification algorithm, drove the real-time game: “jump” a white ball between moving plates (Fig. 8; video clip of real-time play in Supplementary Information). This biofeedback system asks a subject (patient in clinical study) to have “dry swallow” (or other swallowing activities) to jump a ball for continuous traveling (Fig. 8a). The score in the game sum the total number of successful jumping until the ball drops in-between plates. A set of real-time EMG data is summarized in Fig. 8b where the active segments of signals are derived from a “dry swallow” activity. The game control interface concurrently monitors raw and filtered signals from a skin-mounted electrode. The chosen threshold (low and upper) values, based on subject’s training data, determines the active segments of the recorded EMG signals to jump the ball in the feedback game. Each game allows users to play five attempts with fresh balls. When the ball is dropped, the subject has two minutes of rest until next attempt. Note that during the data collection of swallowing EMG signals, it was concluded that any higher swallowing rates more than 3 swallows per minute result in discomfort, which was taken into account when designed the feedback game.

### *In vivo* demonstration of HCI-enabled swallowing training with skin-like electronics

Four subjects (two females and two males) between 21 and 35 years of age in good health (with no reported medical conditions) were recruited to take part in HCI-enabled swallowing training, based on the study protocol (approved number: HM20001454 at Virginia Commonwealth University). The results from this pilot study optimize the device, classification algorithm, and game interface for clinical study with dysphagia patients.

Prior to playing the biofeedback game, we compared the performance between the skin-like and rigid electrodes based on a liquid swallowing test. Table 1 summarizes the classification result with liquid swallowing exercises. The result indicates that the skin-like device shows a similar performance to the rigid one with 100% detection of the swallowing activities. Furthermore, the skin-like electrode shows better false positive rate (3%) than the rigid electrode (5%). Note that false positive was calculated by the number of detection without any observable swallowing activities.

For game control, once skin-like electrodes were placed on submental muscles, two magnitude threshold values were adjusted for each subject to give a satisfactory detection response while observing their swallowing activities. After the calibration, subjects were asked to play the feedback game by dry swallowing. For an assessment of the performance of the skin-like electronics and training game, we extracted the real-time recorded data and compared with recorded videos. This included a total number of attempts with the physical swallowing activities, a number of successful responses, and false positives (number of game responses without any observable swallowing activities). Table 2 summarizes those results from four subjects; the averaged successful response is 94.7 ± 4.4% and 96.2 ± 1.0% for skin-like electrodes and rigid electrodes, respectively. The false positive rate (total false positives per total attempts) for skin-like device is 16.8%, which is slightly lower than the one for rigid device (22.3%). We observed that during the game subjects exaggerated swallowing motions to increase the success rate, which added unexpected muscle activities from tongue and neck/head motions. Those movements increased the false positive rate. In addition, a few missing responses might come from a subject’s impinged swallowing activity to respond to the real-time game play, which sometimes caused weak and/or delayed EMG response.

After the game participation, each subject had an interview to evaluate the acceptability and appropriateness of the skin-like electronics for biofeedback HCI. The evaluation interview included ratings from the lowest 1 to the highest 7 to questions, followed by a description of any thoughts or concerns regarding the soft system. Compared to the rigid electrode (rating basis: 4 out of 7), the skin-like system is highly rated (6.5 in the average out of 7) due to the comfortable interface and perceived acceptance in different environments, which proves the clinical feasibility of this system, together with the results from performance comparison. Specifically, all subjects state that the soft, ultra-light, and stretchable electrode provided negligible effects on the skin, while two subjects express a fragile connection of flexible cables, which slightly lowered the overall rating.

## Discussion

The *in vivo* result with the skin-like electronics showed concerns about the size of the commercial wireless telemetry and interconnecting ribbon cable, which sometimes caused limited mobility. This issue can be resolved by directly incorporating a soft material based wireless circuit with skin-like electrodes. Currently, we study relevant materials and circuit components to design a low-profile, wireless platform by using a strategy of hard-soft materials integration^55^. This type of a total electronic package including soft electrodes would offer an immediate use in extensive clinical study with dysphagia patients. If clinical applications require multiple uses of a skin-like electrode, then a fabric platform with a reversible bonding capability would serve as an alternative (Supplementary Fig. S8). A soft, silky, washable fabric (Enaltus, LLC, Suwanee, GA) with a thin coating of a low-modulus elastomer offers direct mounting of an electrode to the skin without dissolving a thin layer of water soluble membrane. The EMG measurement on the chin shown in Supplementary Fig. S8 demonstrates an intimate contact to the skin, while showing high signal-to-noise ratio, similar to the ultrathin membrane electrode in Fig. 4.

In the study of swallowing activity, targeted signals were detected by using a threshold-based technique. Although this method allowed a signal calibration to overcome variations between users, clear distinction between swallowing and other muscular activities was limited. The result would provide false positives unless users refrained from actions other than the targeted behavior. Adding a trainable classifier would solve this issue by allowing signal responses of interest to be isolated from extraneous muscular activity, caused by other actions. In addition, this technique would further allow to classify a wide range of swallowing patterns, such that users can exercise a set of different activities according to their clinical needs. Another potential solution would be to collect data from other muscle groups during swallowing activity, which will eliminate the signal interference. For instance, data collected from the masseter muscle groups would capture activities during teeth clenching, talking, or swallowing^50^. Inclusion of these data to a trainable classifier might help the detection of swallowing activity from the submental muscles as well as distinguish other additional behaviors.

Future work will include *in vivo* evaluation of the skin-like electronics and HCI training system with patients with dysphagia. The typical characteristics of swallowing EMG signals from patients with dysphagia include slow and reduced laryngeal elevation, long duration of swallow, and slow triggering time of swallow^56^,^57^. Therefore, our classification algorithm, using the three-part threshold technique, should be able to detect swallowing activities from patients after individual calibration of magnitudes and swallowing durations.

## Conclusions

Collectively, we have demonstrated the feasibility of the skin-like electronics for swallowing training in dysphagia rehabilitation via a HCI-based biofeedback system. The soft, conformal electrode enables an ergonomic, high-fidelity recording of swallowing EMG signals, supported by the direct comparison with rigid electrodes. This electrode system demonstrates excellent elastic mechanical compliance in both stretchability (>100%) and bendability (upto 180°), while offering an ideal tissue interface with negligible skin elongation (0.02%) and insensitivity to motion artifacts (~6 times smaller than the conventional one). The game-based biofeedback system finds an important application of the skin-like electronics in swallowing training by motivating users (e.g. patients in clinical study) for repetitive, active swallowing exercises in dysphagia rehabilitation. Areas for further development and future focus include the addition of on-device wireless telemetry and powering system for a portable, home-based rehabilitation.

## Methods

### Analysis of mechanics and materials

Three-dimensional finite element analysis using a commercial software (ABAQUS, Dassault Systems, Waltham, MA) was used to analyze mechanical behaviors of skin-like electrodes upon bending and biaxial stretching. Young’s modulus (*Ε*) and Poisson ratio (*ν*) of materials used in the modeling are *Ε*_*silicone*_ = 69 kPa and *ν*_*silicone*_ = 0.49 for a silicone elastomer (Ecoflex, Smooth-On, Macungie, PA); *Ε*_*Au*_ = 79 GPa and *ν*_*Au*_ = 0.40 for gold nanomembrane; *Ε*_*PI*_ = 2.5 GPa and *ν*_*PI*_ = 0.34 for polyimide (PI; HD MicroSystems, Parlin, NJ). The maximum principal strain applied on the gold membrane was mainly investigated to determine the mechanical stability.

### Fabrication of skin-like electrodes

The details of the step-by-step process are shown in Supplementary Note 1 and Supplementary Figure S9. The fabrication process began with spin-coating of a 100 nm-thick sacrificial layer of polymethylmethacrylate (PMMA; MicroChem, Westborough, MA) and a supporting layer of 1 μm-thick polyimide (PI; HD MicroSystems, Parlin, NJ) on a Si wafer, treated by oxygen plasma (50 W, 20 sccm O_2_ for 30 sec). 300 nm-thick Au membrane was deposited by electron beam evaporation, followed by photolithography and wet etching for electrode patterning. Lastly, the PI layer was patterned based on the same electrode geometry by using reactive ion etching (150 W, 160 mTorr, and 20 sccm O_2_ for 4 min).

### Mechanical stretching and bending test

Biaxial stretching of electrodes was performed with a customized, automated stretcher (details in Supplementary Figure S2). The biaxial stretcher held a sample with four clamps. Control of the travel distance, by an Arduino interface and DC stepper motor, determined the amount of strains. Ultrathin copper wires (100 μm in diameter) were connected to the electrode pads for measurement of resistance change upon applied strains. A digital multimeter (Model 2100, Keithley, Cleveland, OH) was used to measure the signals. In a mechanical bending test, a sample electrode was placed on a flexible plastic film, which allowed mechanical bending from 0 to 180° with the radius of curvature of 500 μm. Similar to the stretching test, electrical resistance was monitored upon the amount of bending. We also utilized a portable USB microscope (Dino-Lite, Torrance, CA) to visually inspect any localized fracture during the mechanical tests.

### Data collection with human subjects

Four subjects (two males and two females), ages from 21 to 35, were given the details of study protocols, used in the swallowing EMG recording and game-based HCI experiment. They all signed a study consent form to participate this study and publication of identifying information/images (when applicable). All *in vivo* tests were conducted at Virginia Commonwealth University, by following the institutional review board approved protocol (HM20001454).

## Additional Information

**How to cite this article:** Lee, Y. *et al*. Soft Electronics Enabled Ergonomic Human-Computer Interaction for Swallowing Training. *Sci. Rep.* **7**, 46697; doi: 10.1038/srep46697 (2017).

**Publisher's note:** Springer Nature remains neutral with regard to jurisdictional claims in published maps and institutional affiliations.

## Supplementary Material

Supplementary Information

Supplementary Video

## Figures and Tables

**Figure 1:**
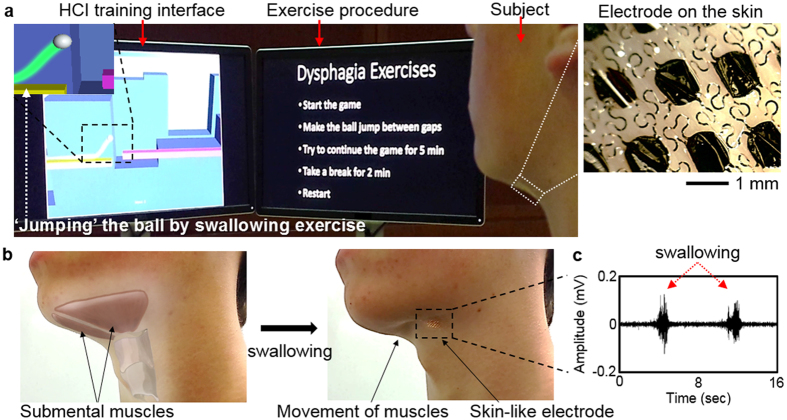
Overview of skin-like electronics enabled HCI for swallowing training. (**a**) Photo of a game-based HCI (‘jumping’ the ball) for swallowing training using skin-like electrodes. (**b**) Illustration of targeted submental muscles on the chin and photos capturing the movement of muscles upon swallowing activity. (**c**) Swallowing EMG signals detected by the skin-mounted electrode in (**b**).

**Table 1:** Performance comparison between skin-like and rigid electrodes.

Subject	Total attempts (Swallows)	Skin-like electrode		Rigid electrode	
		Successful detection	False positives	Successful detection	False positives
1	15	15	0	15	1
2	15	15	1	15	2
3	15	15	1	15	0
4	15	15	0	15	0

**Table 2:** Summary of the classification evaluation results from four subjects.

Subject	Skin-like electrode			Rigid electrode		
	Attempts (Swallows)	Successful responses	False positives	Attempts (Swallows)	Successful responses	False positives
1	20	18	4	21	20	5
2	20	20	0	23	22	8
3	27	26	6	26	25	5
4	52	48	10	42	41	7
