# Supplementary material for: Soft Electronics Enabled Ergonomic Human-Computer Interaction for Swallowing Training
Source: Sci Rep. 2017 Apr 21;7:46697. doi: 10.1038/srep46697 (PMC5399368; doi:10.1038/srep46697)
Supplement: Supplementary Information [file srep46697-s1.pdf]

**Supplementary Information**

# Soft Electronics Enabled Ergonomic Human-Computer Interaction for Swallowing Training

Yongkuk Lee[1,+], Benjamin Nicholls[2,+], Dong Sup Lee[1], Yanfei Chen[3], Youngjae Chun[3,4], Chee Siang Ang[2] & Woon-Hong Yeo[1,5,*]

[1]Department of Mechanical and Nuclear Engineering, School of Engineering, Virginia Commonwealth University, Richmond, VA 23284, USA.

[2]School of Engineering and Digital Arts, University of Kent, Canterbury, United Kingdom.

[3]Department of Industrial Engineering, University of Pittsburgh, Pittsburgh, PA 15261, USA.

[4]Department of Bioengineering, University of Pittsburgh, Pittsburgh, PA 15261, USA.

[5]Center for Rehabilitation Science and Engineering, School of Medicine, Virginia Commonwealth University, Richmond, VA 23284, USA

[+]These authors contributed equally to this work.

*****Correspondence and requests for materials should be addressed to W.-H.Y. (email: whyeo@vcu.edu)

**Supporting Note 1.**

Fabrication process of skin-like electrodes including conventional microfabrication techniques and processes of double transfer printing.

a) Preparing of a carrier wafer

1. Clean a silicon wafer with acetone, IPA, and DI water.
2. Dehydrate it on a hot plate at 110 °C for 3 min.
3. Apply $O_2$ plasma on it at 50 W for 60 sec.
4. Spincoat PMMA on it at 2000 rpm for 30 sec.
5. Bake it on a hot plate at 180 °C for 2 min 30 sec.
6. Spincoat polyimide (PI) at 4000 rpm for 1 min.
7. Pre-bake it on a hot plate at 150 °C for 5 min.
8. Hard bake it on a hot plate at 250 °C for 40 min.

b) Material deposition and photolithography

1. Deposit 300 nm-thick gold (Au) using sputtering systems.
2. Spincoat photoresist (AZ 4620) at 2000 rpm for 30 sec.
3. Bake it on a hot plate at 110 °C for 5 min.
4. Align it with a photomask and expose UV light for 20 sec.
5. Develop patterns with a developer (AZ 400K , 1:3 dilution).
6. Etch Au using gold etchant.
7. Remove photoresist using acetone.
8. Etch PI using reactive ion etcher (RIE) at 150 W, 160 mTorr, and 20 sccm oxygen for 240 sec.

c) Preparation of a thin elastomeric membrane on PVA (polyvinyl alcohol; Haining Sprutop Chemical Tech, China)

1. Tape PVA onto a glass (75 x 50 mm)
2. Prepare 1:2 Ecoflex (part B is 2) and spincoat at 3000 rpm for 120 sec.
3. Cure it on a hotplate at 50 °C for 5 min.

d) Pick up and transfer printing of skin-like electrode onto the silicone/PVA

1. Immerse fabricated skin-like electrodes on a wafer in acetone for overnight
2. Pick up the skin-like electrodes using water-soluble tape
3. Deposit Cr (5 nm)/$SiO_2$ (50 nm) on the skin-like electrodes using an electron beam evaporator
4. Apply $O_2$ plasma on the silicone/PVA at 50 W for 30 sec.
5. Transfer the skin-like electrodes on the silicone/PVA substrate
6. Dissolve the water-soluble tape by gently applying water
7. Bonding a thin, flexible ribbon cable.

**Supplementary Table 1**

**Table S1. Comparison of classification performance between three-part threshold and single-threshold techniques.**

| | | Three Thresholds | | Single Threshold | |
|---|---|---|---|---|---|
| Subject | Total attempts (Swallows) | Successful detection | False positives | Successful detection | False positives |
| 1 | 15 | 15 | 0 | 15 | 7 |
| 2 | 15 | 15 | 1 | 15 | 2 |
| 3 | 15 | 15 | 1 | 15 | 3 |
| 4 | 15 | 15 | 0 | 15 | 0 |

**Supplementary Figure 1**

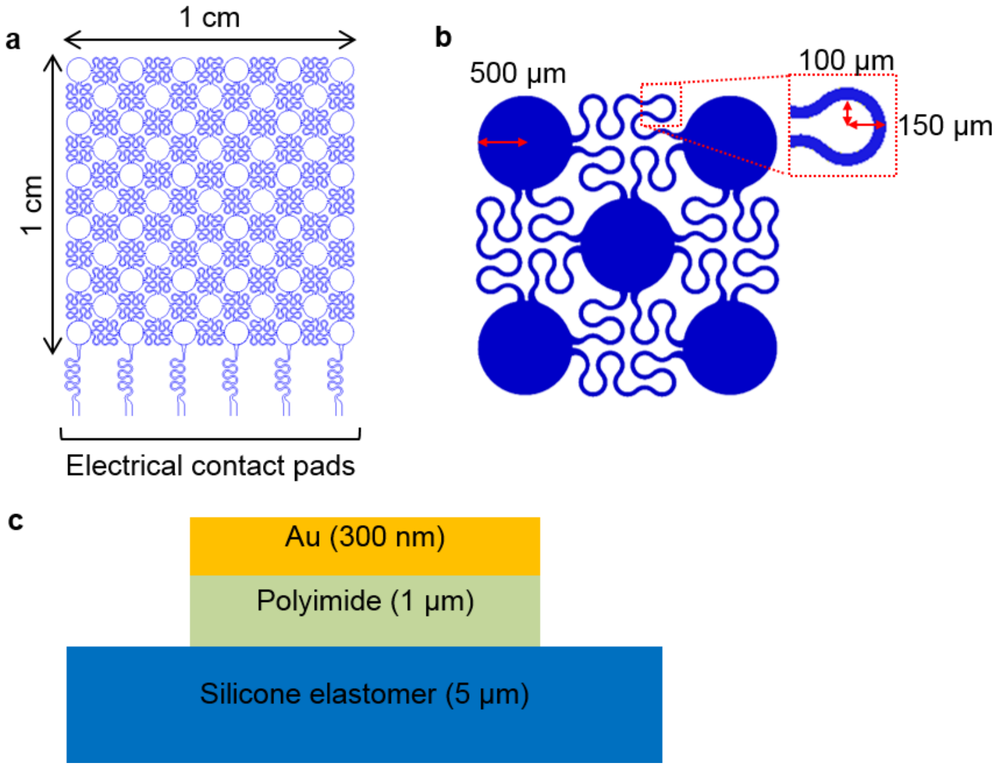


**Figure S1. The design and structure for skin-like electrode for swallowing training in dysphagia rehabilitation.** (a) Dimension of a skin-like electrode. (b) Close-up view of the electrode including circular cells (radius = 500 μm) and fractal interconnects (width = 50 μm). (c) Layer composition of the electrode.

**Supplementary Figure 2**

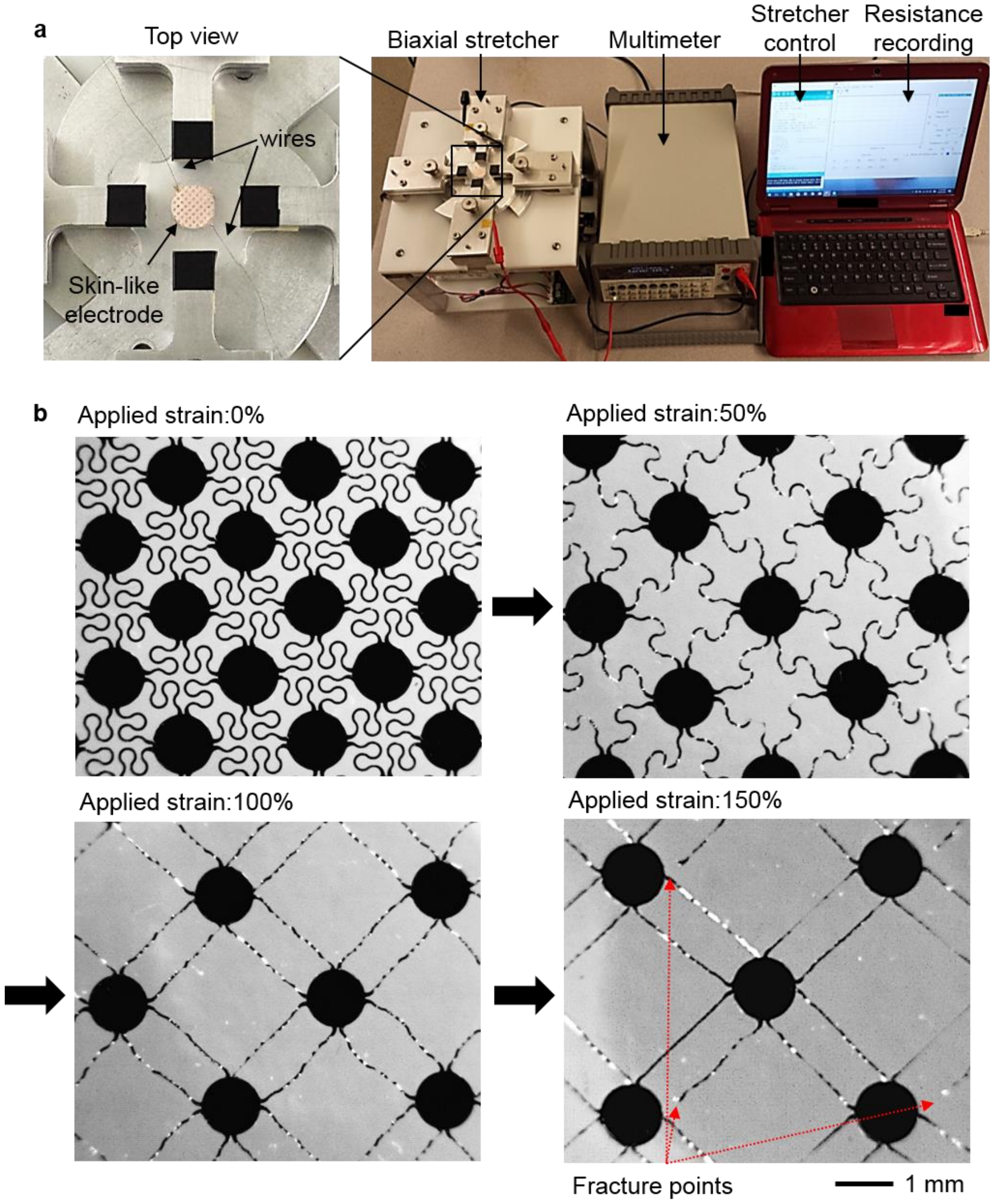


**Figure S2.** (a) Photo of an automated mechanical stretcher (top view) that places a sample in the middle. The biaxial stretcher, controlled by Arduino interface, can precisely apply strains to the sample, while a digital multimeter records the change of electrical resistance. (b) A series of microscopic images of a skin-like electrode with applied strains of 0, 50, 100, and 150%. The first fracture is observed in the structure when stretched up to 150%.

**Supplementary Figure 3**

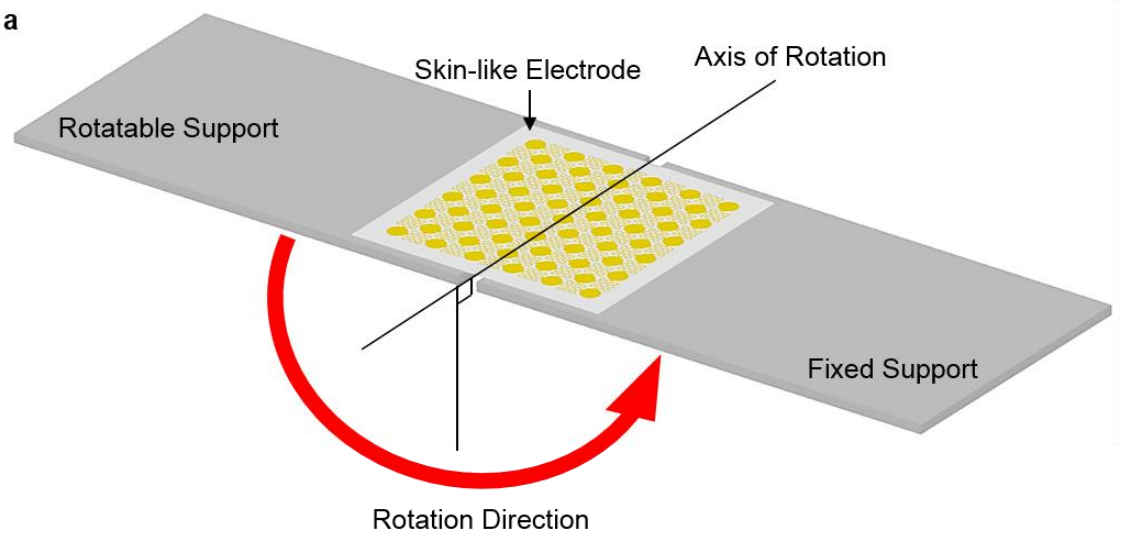


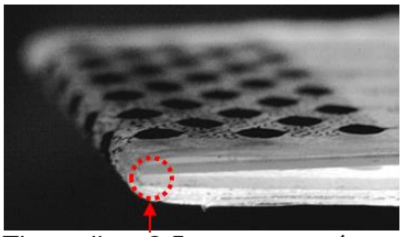


c

| Bending Test | |
|---|---|
| Min Resistance (Ω) | 7.53 |
| Max Resistance (Ω) | 7.67 |
| Change in Resistance (Ω) | 0.14 |

**Figure S3.** Mechanical bending test. (a) Experimental setup for a bending test with the rotation from 0 to 180°. (b) Optical image of a skin-like electrode with bending of 180°. The radius of the curvature is 0.5 mm. (c) Summary of the bending test showing the change of electrical resistance with the maximum bending is negligible.

**Supplementary Figure 4**

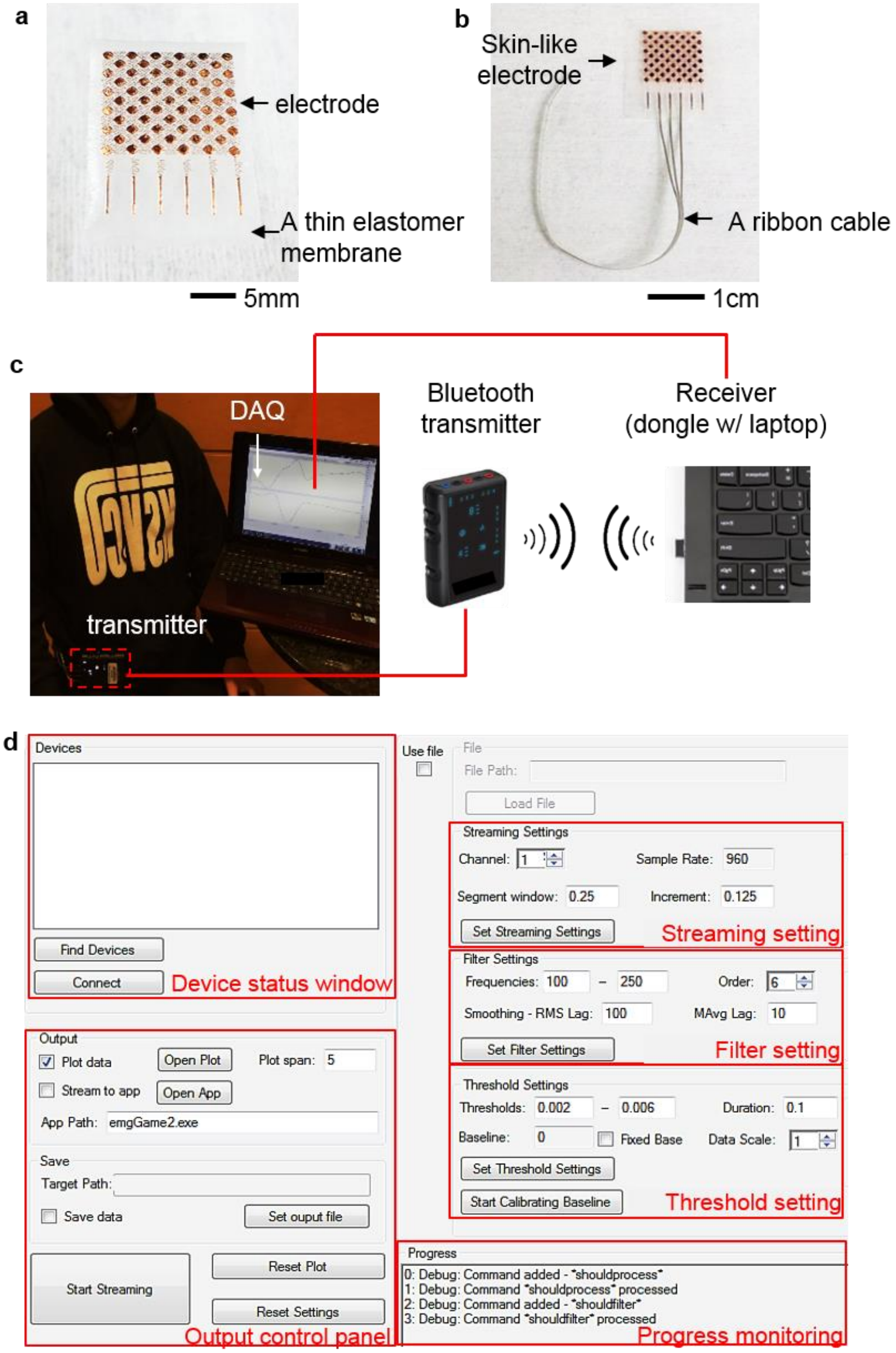


**Figure S4.** (a) Skin-like electrode on a thin transparent elastomer. (b) Skin-like electrode attached with a flexible ribbon cable, which connects the electrode to a wireless recording device. (c) Bluetooth-based wireless transmitter connecting the electrode with the data acquisition system (DAQ) including a wireless receiver (dongle) and data processing software. (d) Photo of software interface.

**Supplementary Figure 5**

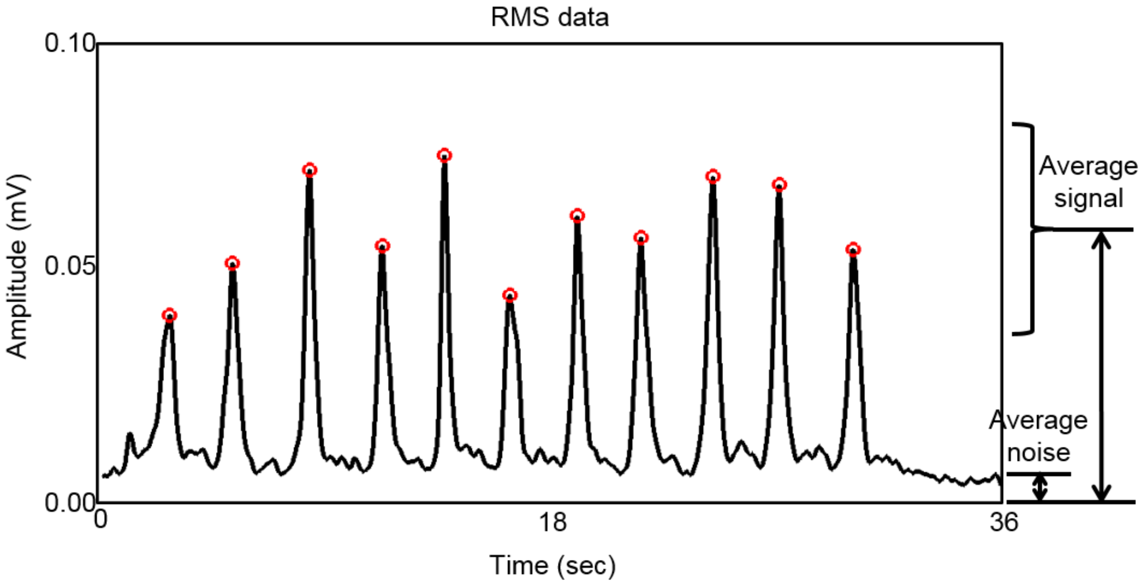


**Figure S5.** Measurement of average SNR values from swallowing EMG signals.

**Supplementary Figure 6**

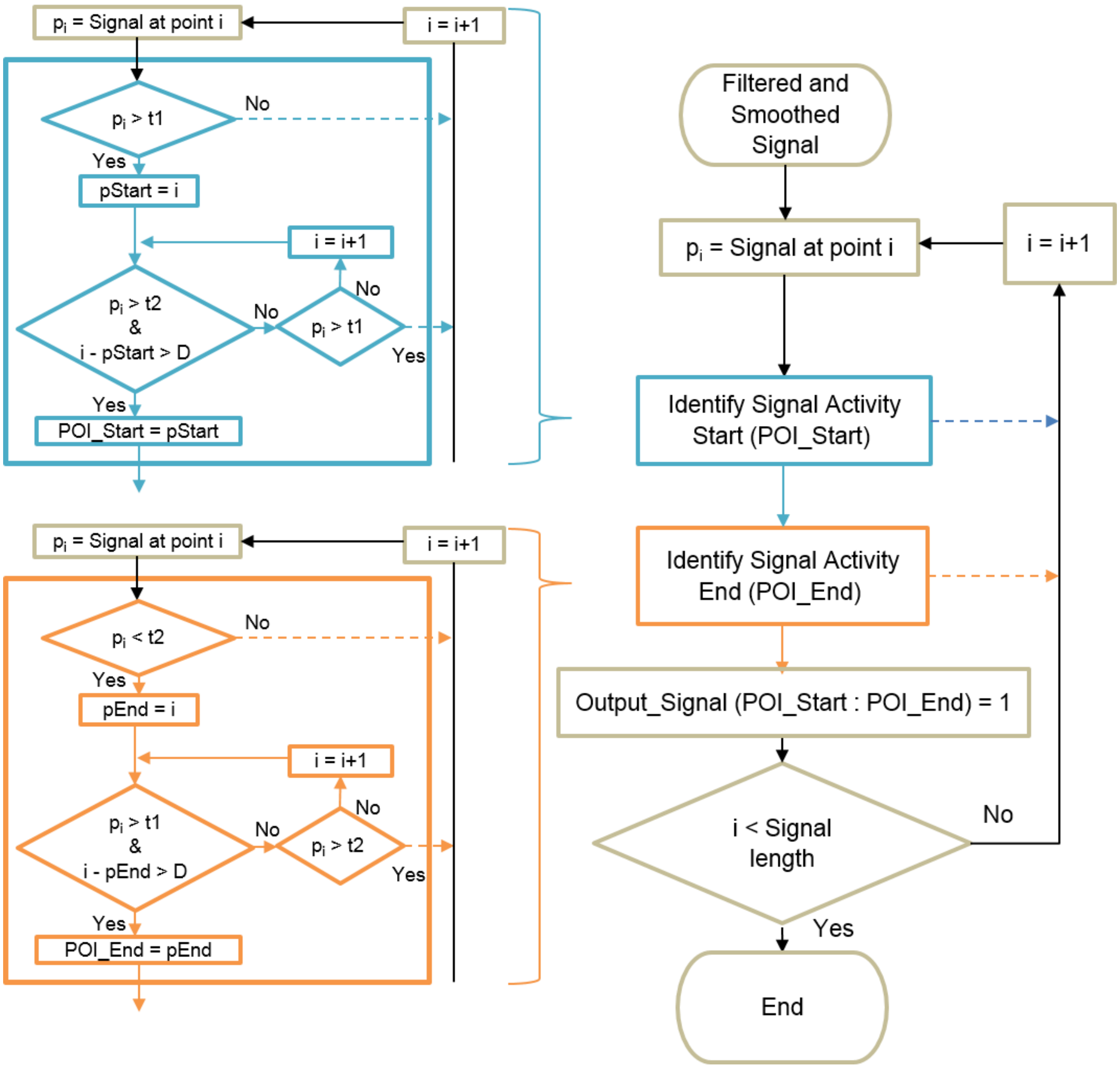


**Figure S6.** A flow chart of the classification algorithm using the three-part threshold technique. The classification algorithm detects the start point of the activy signal segment which exceeds two magnitude thresholds (t1 and t2) and a duration threshold (D) (blue). Finally, the algorithm defines the active signal segment as finding its end point (orange). p = magnitude of the EMG signal; t1 = lower magnitude threshold; t2 = upper magnitude threshold which is larger than t1 and used to ensure a characteristic signal magnitude spike occurs during the active signal segment; i = EMG signal points; D = the duration threshold which is a minimum period of the signals to be an active signal segment.

**Supplementary Figure 7**

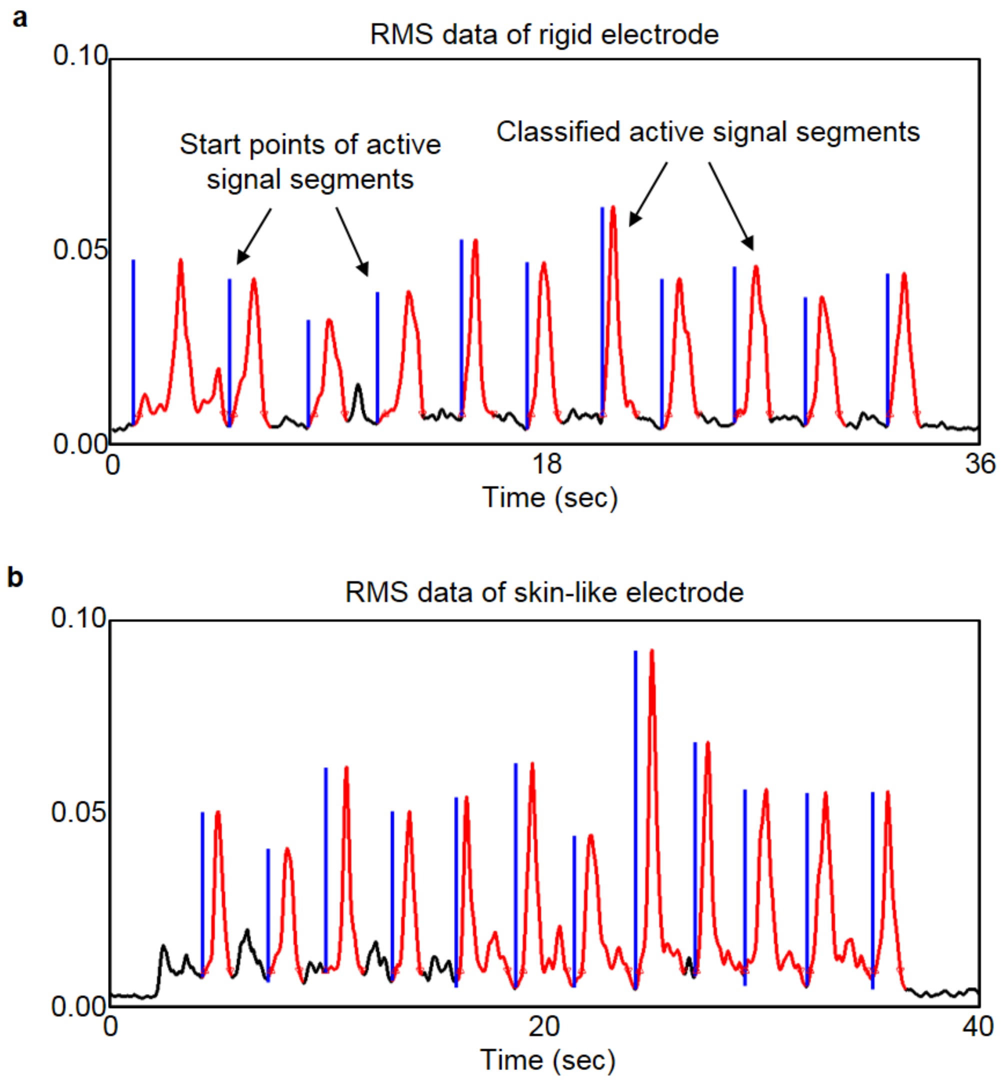


**Figure S7.** Classification results for swallowing RMS data acquired from rigid electrodes (a) and skin-like electrodes (b).

**Supplementary Figure 8**

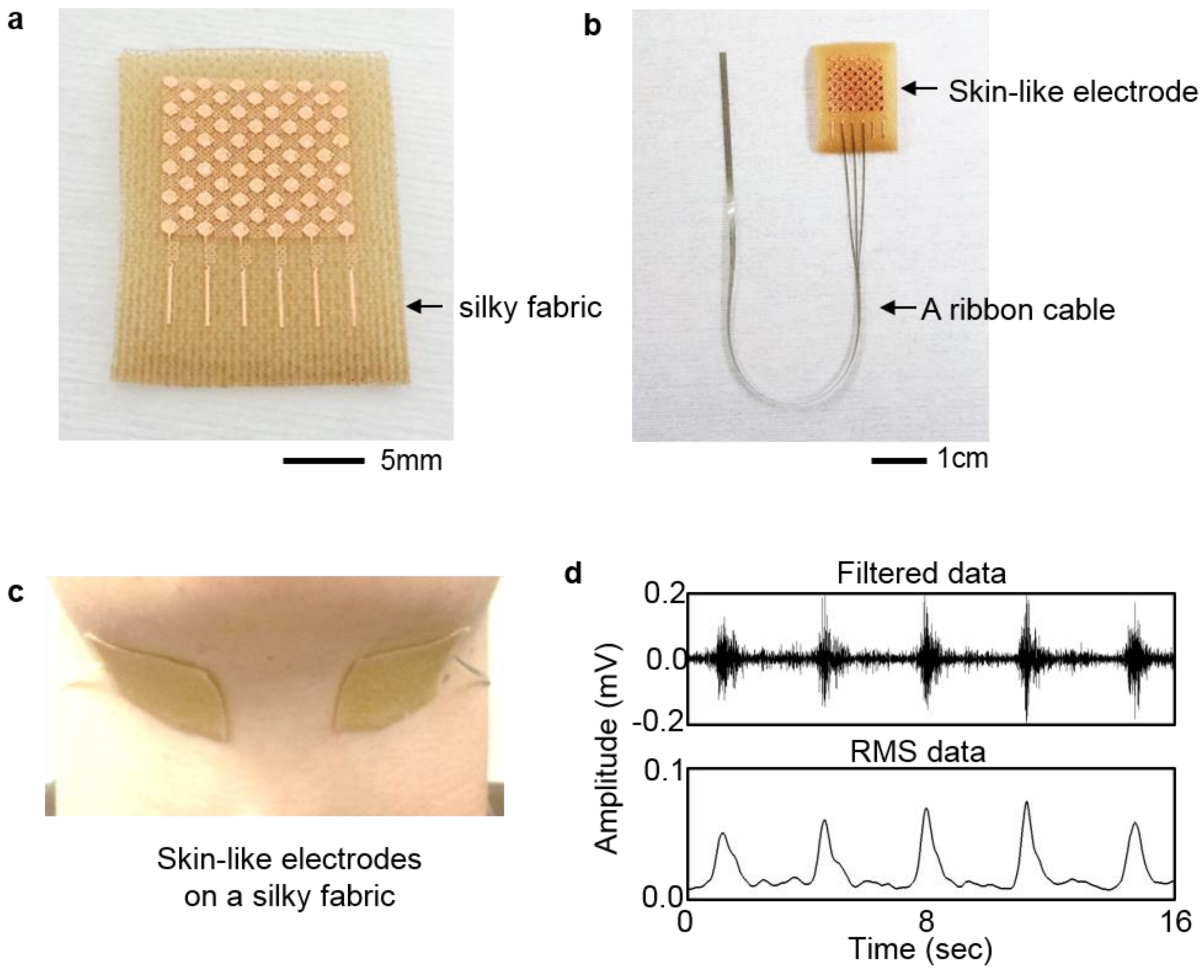


**Figure S8.** (a) Skin-like electrode on an elastomer/silky fabric. (b) Skin-like electrode on a silky fabric attached with a flexible ribbon cable, which connects the electrode to a wireless recording device. (c) Skin-like electrodes on a silky fabric mounted on the neck (submental muscles). (d) Swallowing EMG signals acquired from the skin-like electrodes.

**Supplementary Figure 9**

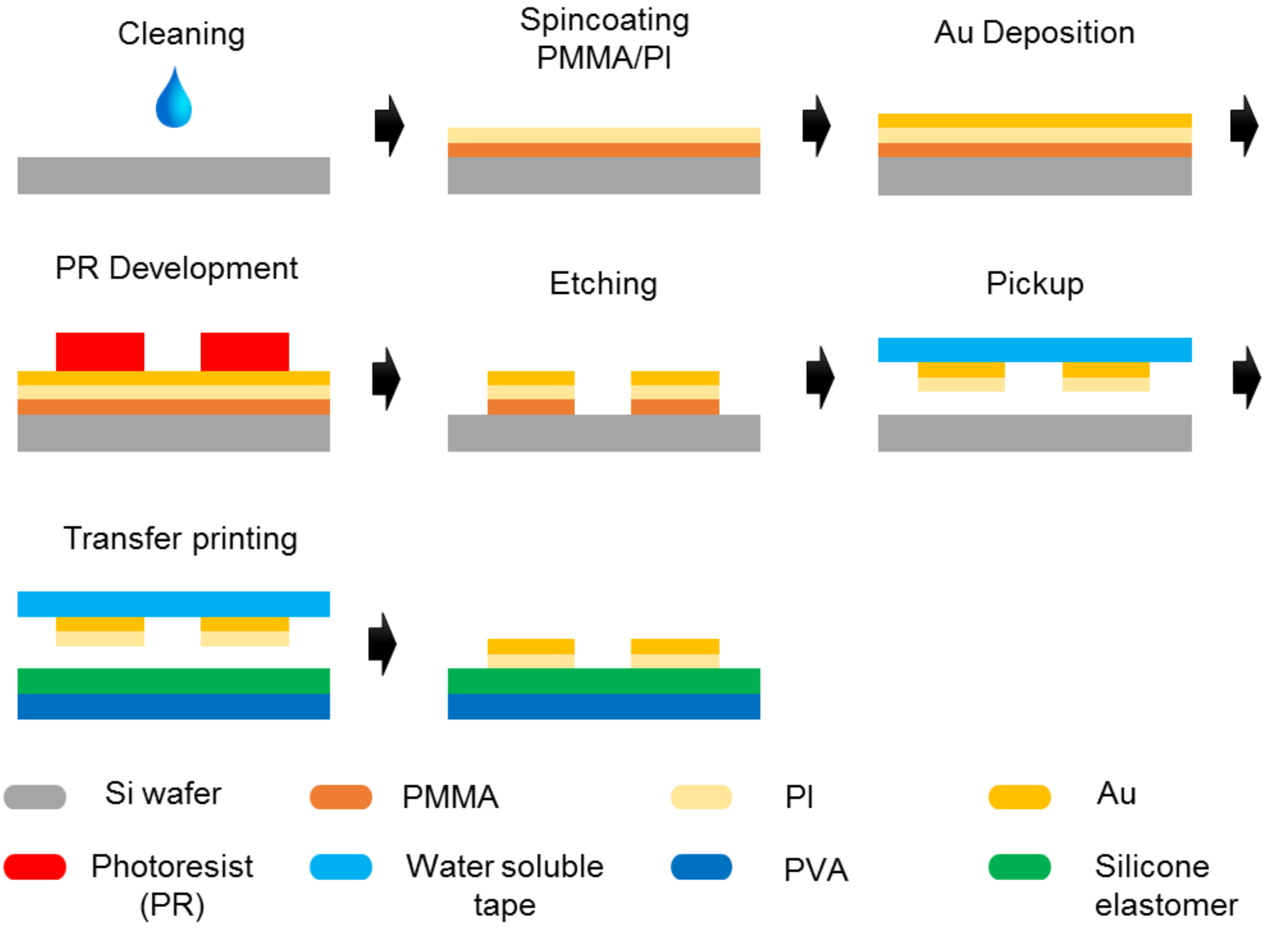


**Figure S9.** Fabrication process of skin-like electrodes.

**Supplementary Video**

Real-time play of a biofeedback game: swallowing training by “jumping” a white ball between moving plates.
